# Data for Psychological Research in the Educational Field: Spotlights, Data Infrastructures, and Findings from Research

**DOI:** 10.5334/jopd.105

**Published:** 2023-12-28

**Authors:** Sonja Bayer, Katarina Blask, Timo Gnambs, Malte Jansen, Débora B. Maehler, Alexia Meyermann, Claudia Neuendorf

**Affiliations:** 1 DIPF | Leibniz Institute for Research and Information in Education, DE; 2 Leibniz Institute for Psychology (ZPID), DE; 3 Leibniz Institute for Educational Trajectories, DE; 4 Institute for Educational Quality Improvement (IQB), DE; 5 Leibniz Institute for the Social Sciences, DE; 6 University of Potsdam, DE

**Keywords:** Psychological Educational Research, Research Data Infrastructure, Data Reuse Challenges, Research Data Center

## Abstract

In recent years, there has been a growing emphasis on the importance of open data and data sharing in scientific research (Nosek et al., 2015; van der Zee & Reich, 2018). However, in the educational field, access to FAIR (findable, accessible, interoperable, and reusable) data remains a significant challenge (Wilkinson et al., 2016). This special collection addresses this challenge by highlighting psychological data in educational research and showcasing examples of data that have been shared and made available to the scientific community in accordance with FAIR principles. With this special collection, we aim to explicitly encourage the use of shared research data for individual research projects.

Shared research data are considered to drive efficiency, knowledge generation, and scientific transparency (Allen & Mehler, 2019; Bainter & Curran, 2015; Nosek et al., 2015; Pontika et al., 2015; Raffaghelli & Manca, 2019; van der Zee & Reich, 2018). It has been pointed out that the effectiveness, productivity, and reproducibility of scientific results are closely linked to the sharing and reuse of research data (Gregory et al., 2020). Researchers can address new research questions with published data. This approach allows them to build on previous studies and explore new areas of inquiry. Moreover, researchers can replicate existing research and its results – either directly, by following the original study’s methods as closely as possible to arrive at similar results, or conceptually, by examining the theoretical soundness of a particular finding or set of findings (Makel et al. 2021; Munafò et al., 2017). In addition, the open accessibility of data facilitates data-driven research and enables analysis of vast amounts of data (see the context of big data). This enhances not only research efficiency but also the potential for new discoveries and insights.

Although replication studies are rare in psychology, the education sciences, and related research fields (ranging from 0.13% in the education sciences to 1.07% in psychology; Makel & Plucker, 2014, 2015; Makel et al., 2012, 2016; Pridemore et al., 2018), this approach is as important as creating and testing innovative new hypotheses. Successful replication of research provides practitioners and policymakers with the confidence to invest resources in interventions derived from that research. Conversely, if research results cannot be replicated, the unnecessary expenditure of further resources can be prevented (Plucker & Mackel, 2021). In cases where prior research cannot be replicated, this can also provide valuable insights into research and research processes in order to identify boundary conditions and further develop theories (e.g., Bryan et al., 2021). Moreover, open data can be used to evaluate the robustness and generalizability of research results by applying multiple analysis methods to a given dataset and using multiple datasets for a given analysis routine (Jansen et al., 2021).

According to Molloy (2011), the more data that are available, the greater the level of transparency and reproducibility in research. Additionally, research output increases when data are shared and well-documented. This is made evident by examining secondary research of data from established international large-scale assessments, such as the *Programme for International Student Assessment* (PISA), the *Trends in International Mathematics and Science Study* (TIMSS), or the *Programme for the International Assessment of Adult Competencies* (PIAAC), which provides public use files and analysis tools for the scientific community. The *International Association for the Evaluation of Educational* Achievement (IEA), for instance, lists more than 160 research papers based on the TIMSS 2015 database (ILSA Gateway, 2023). However, despite the benefits of open data, there are still significant barriers to accessing FAIR data in the educational field (Bainter & Curran, 2015). Studies have shown that the potential of accessible research data has not yet been fully realized (Fecher et al., 2015; Zuiderwijk et al., 2020). For instance, a study on researchers’ perspectives on data reuse found that approximately 40% of researchers in social, behavioural, and economics fields reported difficulties in finding suitable data for secondary analysis, for example because available data did not match the research question, the target population was inappropriate, or researchers did not know where to look for data (Bayer et al., 2022).

Efforts to overcome these challenges are supported by the expanding research data infrastructure. Repositories such as Zenodo or the Open Science Framework (OSF) offer low-threshold opportunities to share data across disciplines and locations. Data can be shared quickly, without cost, and without requirements to comply with format, preparation, and presentation standards. In addition, research data centres (RDCs) offer numerous services that go beyond the SharePoint functionality of repositories to share and make data discoverable. Common services include in-depth documentation of data and assessment processes, information on how to use data, as well as tools and code for data analysis. The underlying aim is to foster data use and support high-quality data analysis.

There are national cross-disciplinary RDCs, like the UK Data Service (UKDS), and discipline-specific RDCs, like the Inter-university Consortium for Political and Social Research (ICPSR) in the USA, GESIS – Leibniz Institute for the Social Sciences in Germany, and FORS, the Swiss Centre of Expertise in the Social Sciences (FORS) in Switzerland. Furthermore, subject-specific RDCs offer specific support for their target groups. The RDC of the Leibniz Institute for Educational Trajectories (RDC LifBi), the RDC for the Programme for International Assessment of Adult Competencies PIAAC (RDC PIAAC), and the RDC at the Institute for Educational Quality Improvement (FDZ at the IQB) provide support in relation to large-scale assessments in education. The Research Data Centre for Education (FDZ Bildung) at DIPF | Leibniz Institute for Research and Information in Education in Germany focuses on qualitative research in education, while the Leibniz Institute for Psychology (ZPID) in Germany provides comprehensive support for psychological researchers. To assist researchers in the education sciences in finding available data across disciplines and selected countries, the German Network of Educational Research Data (VerbundFDB) offers an overarching search functionality.

The datasets presented in this special collection originate in part from this network. The data papers describe accessible data with a broad potential for research and reuse for psychological research in the educational field. Following FAIR principles, the datasets are discoverable and accessible via Digital Object Identifiers (DOIs) that direct researchers to the archiving institutions and to access formalities. They are also interoperable and reusable, which means that data can be processed with common standards and contextual information, thus ensuring high-quality documentation. This special collection comprises 13 data papers presenting quantitative studies. The target populations range from newborns to adults up to age 67. The topics covered include (a) school education and transitions to employment, vocational education, or higher education; (b) the integration of refugee children and adolescents into the education system; (c) factors explaining social inequalities across the life course; (d) Internet addiction, mental health, and maths and statistics anxiety among college students; (e) teacher education; and (f) adult skills and related factors.

Table 1 provides an overview of the topics, target populations, and other details of the studies described in the data papers included in the special collection *Data for Psychological Research in the Educational Field*. The papers in the table are ranked by the age range of the target population.

**Table 1:** Overview of the data papers in the special collection.


NO	AUTHORS	TITLE	KEYWORDS	SAMPLE	SURVEY TIME PERIOD	DATA PAPER	DATA ACCESS

1	Attig et al.	Education from the crib on: The potential of the Newborn Cohort of the German National Educational Panel Study	Newborn cohort Competence development Longitudinal	Newborns and their families (*N* = 3.500)	2012–2022	https://doi.org/10.5334/jopd.81	https://doi.org/10.5157/NEPS:SC5:17.0.0

2	Rohm et al.	Data from the German TwinLife Study: Genetic and social origins of educational predictors, processes, and outcomes	Social inequality Genetic and environmental factors	Same-sex twins of four age cohorts (*N* = 4,096) Parents and siblings	2014–2024	https://doi.org/10.5334/jopd.78	https://doi.org/10.4232/1.13987

3	Thums et al.	Data from the National Educational Panel Study (NEPS) in Germany: Educational pathways of students in Grade 5 and higher (Starting Cohort 3)	Education, competences Secondary school Longitudinal	Grade 5 students (*N* = 6,112) Parents Principals	2010–2022	https://doi.org/10.5334/jopd.79	https://doi.org/10.5157/NEPS:SC3:12.0.0

4	Busse et al.	IQB Trends in Student Achievement 2018: A large-scale educational assessment study in Germany	Large-scale assessment Math and science achievement Monitoring and policy reporting	Grade 9 students (*N* = 51,511) Teachers (*N* = 5,026) Principals (*N* = 1,264)	2018	https://doi.org/10.5334/jopd.71	https://doi.org/10.5159/IQB_BT_2018_v1

5	Kastberg et al.	Data from the Program for International Student Assessment Young Adult Follow-up Study (PISA YAFS): 2012–2016	PISA 2012; follow-up Secondary education Employment	Grade 9 students (age 15; *N* = 2,318) Young adults (age 19)	2012–2016	https://doi.org/10.5334/jopd.82	https://nces.ed.gov/pubsearch/pubsinfo.asp?pubid=2021022

6	Hupka-Brunner et al.	Data from the Swiss TREE panel study(Transitions from Education to Employment)	School-to-work transition Educational & occupational trajectories Life course research	Grade 9 students (*N*_max_ = 8,428) Young adults (age 30)(*N*_max_ = 4,461)	2000–2020,2016–2022	https://doi.org/10.5334/jopd.97	https://doi.org/10.23662/FORS-DS-816-7 https://doi.org/10.48573/kz0d-8p12

7	Heers et al.	Data from the mixed method project PICE (Parental Investment in Children’s Education)	Parental investment Migration Mixed methods	Young adults (*N* = 73, around age 20) Their parents (*N* = 50)	2019–2022	https://doi.org/10.5334/jopd.95	https://doi.org/10.48573/hjc1-3171

8	von Maurice & Will	Data from the panel study “Refugees in the German Educational System (ReGES)”	Refugees Educational transitions Integration	Refugee children (aged 4 years and above but not yet attending school; *N* = 2,405) and adolescents (age 14–16; *N* = 2,415)	2018–2020	https://doi.org/10.5334/jopd.77	https://doi.org/10.5157/ReGES:RC1:SUF:3.0.0 https://doi.org/10.5157/ReGES:RC2:SUF:3.0.0

9	Schaeper et al.	The German Panel of Teacher Education Students: Surveying (prospective) teachersfrom higher education into working life	Teacher education Preparatory service Panel study	(Prospective) teachers(*N* = 2,757–1,388)	2010–2022	https://doi.org/10.5334/jopd.76	https://doi.org/10.5157/NEPS:SC5:17.0.0

10	Martin et al.	Data from PIAAC Germany and its longitudinal follow-up, PIAAC-L	Cognitive skills Large-scale assessment Longitudinal survey	Adults (age 16–67; *N* = 5,465) Spouses/partners (*N* = 1,539) Household members (*N* = 934)	2011–2012,2014–2015, 2016	https://doi.org/10.5334/jopd.74	https://doi.org/10.4232/1.12660

11	Buddeberg et al.	Data from LEO 2018 – Living with Low Literacy	Competences and background Digital and writing practices Political participation	Adults (age 18–64; *N* = 7,192)	2018	https://doi.org/10.5334/jopd.91	https://search.gesis.org/research_data/ZA6266 https://search.gesis.org/research_data/ZA6265

12	Mwakilama et al.	Data from “Internet Addiction and Mental Health among College Students in Malawi”	Mental health Internet addiction Anxiety	College students (age 15–30; *N* = 984)	2018	https://doi.org/10.5334/jopd.72	https://doi.org/10.17632/xbfbcy5bhv.3

13	Terry et al.	Data from an international multi-centre study of statistics and mathematics anxiety and related variables in university students (the SMARVUS dataset)	Anxieties (e.g. maths, test, social interaction, creativity) Self-efficacy Higher education	Students (age 18–67; *N* = 12,570) 100 universities; 35 countries	2021	https://doi.org/10.5334/jopd.80	https://doi.org/10.17605/OSF.IO/MHG94


The database *(1)* presented by Attig et al. (2023) covers the Newborn Cohort of the German National Educational Panel Study (NEPS SC1) and provides longitudinal data on 3,500 newborns and their families. The overarching goal of NEPS is to collect high-quality longitudinal data to investigate research questions on competence development and educational processes in Germany across the lifespan. The datasets of the nine waves of the NEPS SC1 survey published to date can be accessed for scientific use via the RDC LIfBi. The NEPS SC1 database includes data from standardised and semi-standardised observation measures and competence tests for children, as well as interviews and questionnaires for parents or caregivers. This is a rich source of data that can be used to analyse educational trajectories from infancy onwards, including the analysis of influencing conditions, factors, and educational processes that are important for explaining individual differences in child development.

The contribution by Rohm et al. (2023) describes an 8-year longitudinal study *(2)* – the German Twin Family Panel (TwinLife) – that began its data collection when the children were 5 years of age. Focusing on social inequalities and educational processes, the study follows the families of four age cohorts of same-sex twins across childhood, adolescence, and young adulthood. The objective of the study was to investigate the interplay of genetics and environment in explaining social inequalities across the life course. The survey period includes the years of the COVID-19 pandemic, with supplementary surveys capturing in more detail the impact of this period on the lives of young persons. The TwinLife study incorporates a large variety of psychological constructs (e.g., motivation, personality, socio-emotional well-being, and self-regulation), a cognitive ability test, educational biographies, family background and home environment, as well as the possibility to work with genetic data collected with saliva samples. With its design, the TwinLife study offers unique opportunities to study genetic and environmental influences on educational development and career paths.

In addition to collecting data from the NEPS Newborn Cohort (see Attig et al., 2023, in this special collection), the longitudinal NEPS study also gathers data *(3)* on the educational pathways of fifth-grade students in Germany (NEPS SC3), which are described in the contribution by Thums et al. (2023). This database includes annual student interviews, competence tests, and information on important attachment figures such as parents, teachers, and school administrators. A wide range of psychological constructs are incorporated into the data, such as socio-emotional competencies; motivation; mental health; domain-general competencies; domain-specific competencies, such as reading, maths, and science; and meta-competencies. As a result, this database serves as a comprehensive foundation for research in developmental and educational psychology.

The next two papers in this special collection present data from ninth-grade students in Germany: The 2018 IQB Trends in Student Achievement Study *(4)* described by Busse et al. (2022) focuses on students’ education and learning outcomes in Germany. The IQB Trends in Student Achievement Study is a large-scale, nationally representative educational assessment that is conducted at regular intervals and is fundamental to educational monitoring in Germany. The data are accessible through the research data centre (FDZ) at the Institute for Educational Quality Improvement (IQB).

The datasets described by Busse et al. (2022) provide a rich resource on the academic lives of 9th-grade students in Germany, including curricular-valid achievement estimates for mathematics and science, general cognitive abilities, motivational, socio-emotional and whole-classroom social network data, as well as information about students’ socio-economic background, their classrooms, and schools. The structure and size of the study provide unique opportunities for secondary analyses. Multiple informant data exist, for example from teachers, parents, and students. Moreover, because complete school classes were sampled, questions regarding classroom structures and networks can be addressed and contextual effects on students can be researched. Finally, the size of the datasets allows robust analyses with sub-samples, for example refugee students or students with special educational needs. And because the datasets are part of a trend study, cohort comparisons are also possible.

The Program for International Student Assessment Young Adult Follow-up Study (PISA YAFS) described by Kastberg and colleagues (2023) was a follow-up study *(5)* conducted in 2016 in the United States with young adults who had participated in PISA 2012 when they were in high school. The study aimed to measure the relationship between performance on PISA 2012 and subsequent outcomes (e.g., education and employment) as well as skills. Skills were assessed in an online assessment of reading literacy, numeracy, and problem-solving skills called Education and Skills Online (ESO). The study allows research into the characteristics, cognitive skills, and other life outcomes of young adults as they transition from high school to post-secondary life. Other potential explorations include analysing the data for different population groups, such as ethnic groups that had different skills trajectories from those of the overall 19-year-old population.

While the studies mentioned above focus on the educational experiences of secondary school students, the following studies examine transition processes from secondary school and higher education to working life. The contribution by Hupka-Brunner et al. (2023) presents the TREE (Transitions from Education to Employment) study (*6*), which is a prospective interdisciplinary mixed-mode panel study following up on the post-compulsory education and employment trajectories of two Swiss cohorts of compulsory school-leavers. TREE1 is a follow-up survey from PISA 2000. TREE2 started in 2016 and draws on a national large-scale assessment of mathematics skills. The TREE survey programme covers various aspects of the life course, namely educational and employment trajectories, intra-psychic developments, and various areas of life (family and networks, leisure, health, social participation).

The Parental Investment in Children’s Education (PICE) study (*7*) described by Heers et al. (2023) complements the TREE study and investigates parental strategies, resources, and aspirations, and how parents shape their children’s educational pathways. It compares families with and without a migration background. Reuse potential relates, for example, to parental investments and migration biographies. Due to its innovative design, PICE enables specific methodological research on mixed methods.

Addressing refugees’ transition to school, transitions within the general school system, and transitions to the vocational educational system or tertiary education, the contribution by von Maurice and Will (2023) describes longitudinal data *(8)* from the Refugees in the German Educational System (ReGES) study. ReGES adopted a multi-informant perspective and a multi-method approach, including personal interviews and telephone and online interviews. The data can be used to address diverse topics related to the pathways of the refugee population that entered Germany in the mid-2010s, ranging from educational transition paths to individual development paths. Thus, the data can be of great relevance for addressing various issues not only in educational science but also, for instance, in developmental psychology, economics, and sociology.

The contribution by Schaeper et al. (2023) presents longitudinal data of prospective teachers in Germany (*9*) collected within the framework of the German Panel of Teacher Education Students (LAP). The database provides longitudinal information from over 10 years of panel surveys, covering the entire teacher education phase and early years in the teaching profession, with a large sample of prospective teachers in Germany. The database is not limited to specific German federal states, school types, or subjects taught, and can be used for a variety of research questions on teacher education, the transition to the teaching profession, and the first years in the teaching profession. The database can also be used to analyse the educational and occupational trajectories and the professional situation and self-assessed competencies of prospective teachers.

Finally, the special collection on educational data for psychological research includes four data papers describing databases that cover the skills and related factors of adults aged up to 67 years and the mental health and well-being of college students. Martin et al. (2022) present a dataset *(10)* collected in Germany within the framework of the *Programme for the International Assessment of Adult Competencies* (PIAAC) and its longitudinal extension, PIAAC-L. PIAAC is a large-scale assessment initiated by the Organisation for Economic Co-Operation and Development (OECD) that measures the cognitive skills (e.g., literacy, numeracy and problem solving in technology-rich environments) of the adult population. In addition, a background questionnaire collects detailed information about the respondents. PIAAC-L was a three-wave longitudinal extension in which PIAAC respondents and, additionally, their household members and partners were interviewed and followed across three consecutive years. A special feature of these data is their alignment with the questionnaires of the German panel studies NEPS and the Socio-Economic Panel (SOEP), which makes it feasible to use them in the context of an integrative data analysis (Bainter & Curran, 2015). Comprehensive information on the participants’ demographic background, biographies, health, skills, and psychological features lends itself to a variety of research questions on human development that could be answered with these data.

Focusing on adults with low literacy skills in Germany, the study LEO 2018 – Living with Low Literacy (*11*) presented by Buddeberg et al. (2023) examined reading and writing skills. The study included a literacy assessment and an extensive background questionnaire that collected information on participants’ literacy-related everyday practices and domain-specific basic skills (digital, financial, health-related, and policy-related). Furthermore, the study focused on text-related practices in various contexts (work, family, everyday life) and asked about participation in continuing education, migration and multilingualism.

The study *(12)* presented by Mwakilama et al. (2022) examined the relationship between Internet addiction and mental health among students from 13 tertiary education institutions in Malawi. Data were collected from the students using a single structured questionnaire that was designed on Google Forms. The questionnaire included demographic measures, an Internet Addiction Test (IAT), and a Self-Reporting Questionnaire (SRQ-20) to detect probable cases of common mental disorders (CMD). The SRQ-20 includes variables that may indicate probable mental health disorder conditions in relation to Internet addiction. The authors note that the SRQ-20 data “provide an opportunity to draw out anxiety scores related to the Beck Anxiety Inventory (BAI) approach”.

The study *(13)* described in the data paper by Terry et al. (2023) addressed the question of whether statistics anxiety and mathematics anxiety are one and the same construct. It used an experimental design and examined undergraduate psychology students to shed light on this issue. The authors describe the SMARVUS dataset, a large international dataset from an International multi-centre study of statistics and mathematics anxieties and related variables in university students. The study was conducted first to assess the generalisability of construct-validity findings from a study of undergraduate psychology students in the UK by repeating it in a large, international sample of students from various disciplines, and second to further test the construct validity of established measures in this field. The dataset contains survey responses from students at 100 universities across 35 countries for whom statistics was part of their degrees. The data could be reused to address diverse research questions with a special focus on statistics and mathematics education, anxiety, psychometrics, or survey methodology.

In summary, this special collection presents high-quality, cross-sectional, and longitudinal educational databases covering age cohorts from newborns to age 67, including large sample sizes, diverse samples, and a multitude of background information that enables further research. Thanks to policy interventions in research funding, Germany has experienced significant growth in available research data and research data infrastructure in the past decade. This growth is apparent in the data papers in the current special collection, eight of which originate from Germany. However, the collection also includes two data papers that draw on Swiss studies, one data paper focusing on students in Malawi, one data paper describing an international dataset covering 35 countries, and one data paper describing a study conducted in the USA. We eagerly anticipate that this special collection on data for psychological research in the field of education will lead to increased use of these datasets.
